# Modeling and Performance Analysis of Movement-Based Group Location Management Using RFID Sensing in Public Transportation Systems

**DOI:** 10.3390/s121216077

**Published:** 2012-11-22

**Authors:** Yun Won Chung

**Affiliations:** School of Electronic Engineering, Soongsil University, 511 Sangdo-dong, Dongjak-Gu, Seoul 156-743, Korea; E-Mail: ywchung@ssu.ac.kr; Tel.: +82-2-820-0908; Fax: +82-2-821-7653

**Keywords:** radio frequency identification (RFID), location management, paging, group, movement-based, transportation system, cellular system

## Abstract

Location management, which consists of location registration and paging, is essential to provide mobile communication services to mobile stations (MSs). Since MSs riding on a public transportation system (TS) generates significant location registration signaling loads simultaneously when a TS with riding MSs moves between location areas (LAs), group location management was proposed. Under the group location management, an MS performs group registration when it gets on a TS and performs group deregistration when it gets off a TS. Then, only a TS updates its current location when it changes LA, on behalf of all riding MSs. In this paper, movement-based group location management using radio frequency identification (RFID) is proposed, where the MS’s getting on and getting off behaviors are detected using RFID and only location update of a TS is carried out if the number of crossed cells from the last updated cell exceeds a predefined movement threshold, on behalf of all riding MSs. Then, we develop an analytical model for the performance analysis of the movement-based group location management and analyze the effects of various parameters on the performance. The results show that the movement-based group location management has reduced signaling cost compared with movement-based individual location management, and optimal performance can be achieved by choosing appropriate movement threshold values.

## 1. Introduction

In mobile communication systems, location management, which consists of location registration and paging, is essential to provide mobile communication services to mobile stations (MSs) [1]. In location registration, an MS updates its location information at location registers (LRs) when it changes location area (LA) consisting of a group of cells. An incoming call is delivered to a called MS by paging all cells within a registered LA based on the stored location information in LRs. There is a tradeoff relationship between the number of location registration messages and paging signaling messages.

In current cellular systems, static zone-based location registration scheme is widely adopted, where cells are grouped as a fixed LA. However, significant signaling loads are generated at cells located in the boundary of LAs. Also, diverse traffic and mobility characteristics of MSs are not efficiently accommodated in static zone-based location management scheme. Therefore, dynamic location registration schemes, such as timer-based [2,3], distance-based [4–6], and movement-based [7–10] schemes were proposed. In the timer-based location registration scheme [2], an MS performs a location registration periodically with a period of a predefined time threshold. In the distance-based location registration scheme [4], an MS performs a location registration whenever it moves for a predefined distance threshold from the last registered location. In the movement-based location registration scheme, an MS performs a location registration whenever it moves for a predefined movement threshold [7] from the last registered cell. In these individual location management schemes, which manage the location information of an individual MS separately, however, significant simultaneous location registration signaling loads of MSs riding on a transportation system (TS) are generated when a TS with riding MSs moves between LAs [11].

In order to solve the significant signaling load problem of individual location management, group location management was proposed to reduce the significant simultaneous location registration signaling loads of MSs riding on a transportation system (TS) when a TS with riding MSs moves between LAs [11]. Under the group location management, an MS performs group registration when it gets on a TS and performs group deregistration when it gets off a TS. Then, only a TS updates its current location, on behalf of all riding MSs, when it changes LA [11–16].

**Figure 1.**
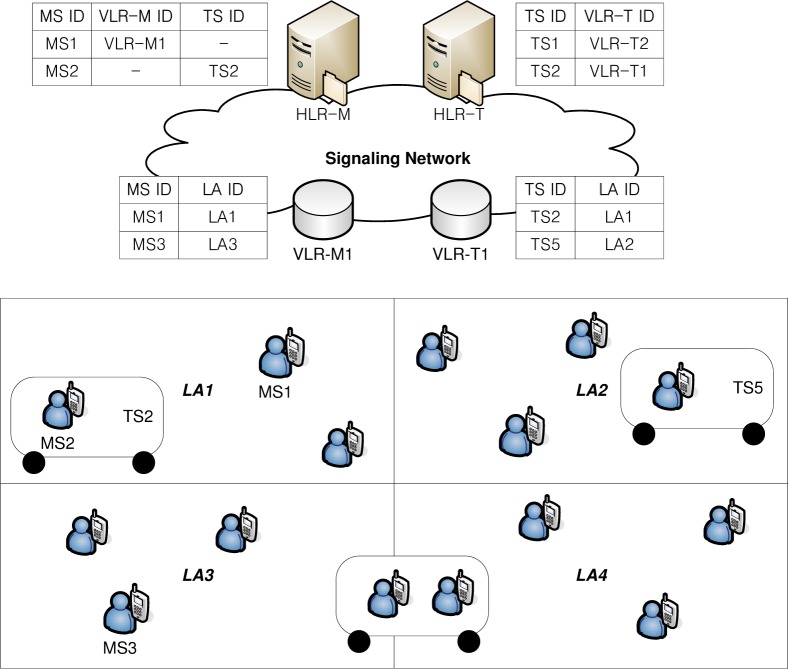
A network architecture for group location management based on zone-based location registration. (©2011 IEEE Reprinted with permission, IEEE/IPSJ 11th International Symposium on Applications and the Internet (SAINT) 2011, [20]).

Figure 1 shows a network architecture for group location management based on zone-based location registration, where cells are grouped into static LAs. In Figure 1, there are four LRs, *i.e.*, home LR for MS (HLR-M), visitor LR for MS (VLR-M), HLR for TS (HLR-T), and VLR for TS (VLR-T). Under the group location management, an MS performs group registration when it gets on a TS, by sending MS identity (ID) and TS ID, which are broadcasted from the base station (BS) installed in a TS [11]. Thus, the relationship between MS ID and TS ID is stored in the HLR-M. After that, the location of MSs riding on a TS is managed by the location of a TS since the MSs riding on a TS have the same mobility characteristics with the TS. Thus, only a single location registration by the TS is sufficient to manage the location information of all riding MSs when the public TS changes LA. This can significantly reduce the location registration signaling load of MSs in the individual location management. When an MS gets off a TS, group deregistration is performed and the relationship between MS ID and TS ID is removed from HLR-M. Instead, the relationship between MS ID and VLR-M ID is stored in HLR-M since the location of an MS is managed by individual location management. In call delivery to an MS riding on a TS, the LRs of the TS, *i.e.*, HLR-T and VLR-T, are queried to find the current LA of the TS, and paging messages are sent to all cells within the registered LA of a TS [11–15]. In call delivery to an MS not riding on a TS, the LA ID of the called MS is obtained by querying VLR-M from HLR-M, and paging messages are sent to all cells within the registered LA of the called MS.

Most previous works on group location management, however, concentrated on static zone-based location registration [11–15]. However, significant signaling loads are generated at the boundary cells of static LAs in zone-based location registration, and diverse traffic and mobility characteristics of MSs and TSs are not efficiently accommodated. Therefore, movement-based location management, which is actively being studied as a representative dynamic location management [17–19], needs to be applied for efficient group location management.

In our preliminary work, we extended the zone-based group location management by considering movement-based location registration [20] and analyzed the performance of movement-based location registration based on simple exponential distribution for cell residence time and the same movement threshold values for both MSs and TSs. In this paper, we extend the work in [20] by developing a more detailed analytical model by assuming general distribution for cell residence time, and Gamma distribution is used in numerical examples. Also, different movement threshold values are assumed for MSs and TSs, respectively, and the effect of different movement threshold values were analyzed in detail.

Furthermore, we propose a way of how to detect the behavior of an MS getting on and getting off a TS by using radio frequency identification (RFID) technology. In previous works on group location management, it is assumed that another BS is installed at a TS and it broadcasts TS ID [11], but it is impractical because significant modification should be made in current cellular network architecture. However, our proposed scheme using RFID technology can be implemented without changing the current cellular network architecture, with the aid of RFID technology. In the proposed scheme, RFID tags with TS ID information are installed inside a TS, and an MS with RFID reader can detect the RFID tag when it gets on a TS and identify the TS ID. RFID tags are detected continuously while the MS stays in the TS. On the other hand, if the MS gets off the TS, RFID tags are not detected. If no RFID tag with the previously identified TS ID is detected until a predefined timer expires, the MS determines that it is now located outside the TS and performs individual location update.

Incorporating RFID reader into an MS is a relatively recent technology than conventional RFID technology and is currently serviced as mobile RFID phone service [21,22]. In Mobile RFID, an MS can identify RFID tag using mobile phone, contrary to the conventional fixed RFID reader, where RFID tags are mobile [23]. New services such as mobile payment, information retrieval, diagnostic sensors, museum guide, wearable devices with RFID reader, *etc.*, can be provided using mobile RFID technology [22,24,25]. In [26], both RFID tags and RFID readers are fixed and the location of elderly people is tracked.

The major contributions of this work are as follows:

- A detailed algorithm of movement-based group location management procedures and corresponding location information registered at LRs are presented.
- A detailed analytical model of MS’s getting on and getting off a TS and location registration by MSs and TSs is developed based on general cell residence times of both MSs and TSs.
- Different movement threshold values of MSs and TSs are assumed and the effect of different movement threshold values is analyzed.
- The signaling loads due to location registration, group registration, and group deregistration are analyzed in numerical examples using Gamma distribution, and the effect of the variance of cell residence times is analyzed.
- The developed model is used to evaluate the performance of the movement-based group location management compared with that of the movement-based individual location management.
- The use of radio frequency identification (RFID) technology is proposed to detect the behavior of an MS getting on and getting off a TS.

The remainder of this paper is organized as follows. Section 2 presents the group location management based on movement-based location registration. The performance of the group location management based on movement-based location registration is analyzed in Section 3. Numerical examples are presented in Section 4. Finally, Section 5 concludes this work and presents our future work.

## 2. Movement-Based Group Location Management Using RFID Sensing

**Figure 2.**
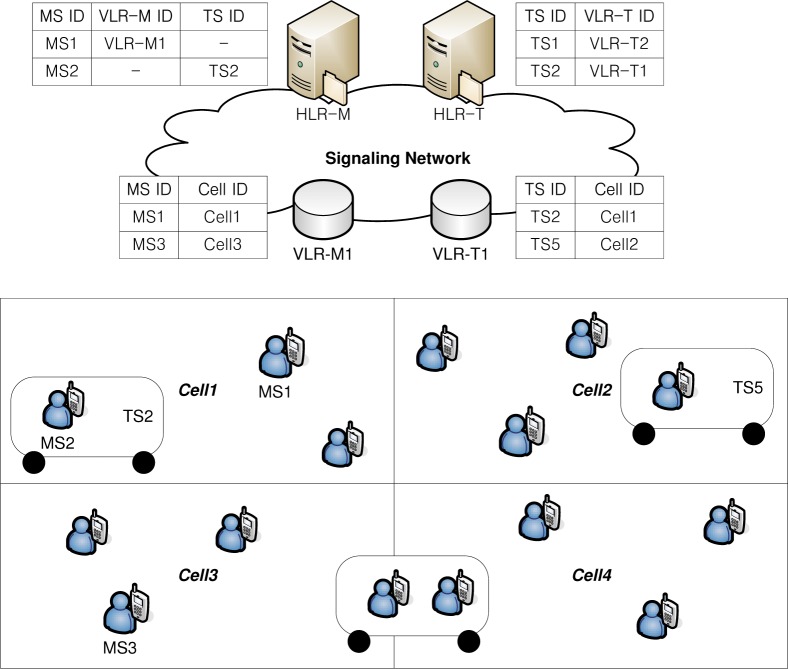
A proposed network architecture for group location management based on movement-based location registration. (©2011 IEEE Reprinted with permission IEEE/IPSJ 11th International Symposium on Applications and the Internet (SAINT) 2011, [20]).

**Figure 3.**
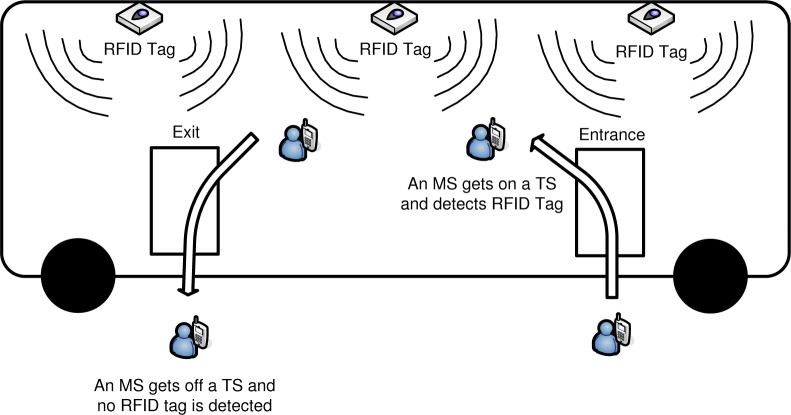
Detection of MS’s getting on/off behavior.

Figure 2 shows a network architecture for the group location management based on movement-based location registration. Contrary to Figure 1, where the LA ID is stored in VLR-M and VLR-T, the cell ID is stored in VLR-M and VLR-T for movement-based location management. In the proposed scheme, we assume that RFID reader is incorporated in an MS. When an MS gets on a TS, it can detect the getting on behavior, since it can detect RFID tags containing the TS ID information attached to a TS, as shown in Figure 3. Also, when an MS gets off a TS, it detects the getting off behavior since the stored RFID tag is not detected anymore. The location information in HLR-M depends on whether an MS is riding on a TS. If an MS is riding on a TS, the relationship between the MS ID and the TS ID where the MS is riding is stored in HLR-M, since an MS performs group registration when an MS gets on a TS. When a TS with MSs moves between cells, the location of the MSs riding on a TS is managed only by a TS and a TS updates its location information whenever the number of crossed cells from the last updated cell exceeds a predefined movement threshold for a TS.

An MS riding on a TS does not need to update its location in the group location management. If an MS is not riding on a TS, the relationship between MS ID and VLR-M ID, which manages the last updated cell ID of the MS, is stored in HLR-M, since an MS performs group deregistration when it gets off a TS. When an MS gets off a TS, since the MS is managed by individual location management, it updates its current cell ID as its last updated cell ID in VLR-M instantly. In HLR-T, the relationship between TS ID and VLR-T ID, which manages the last updated cell ID of the TS, is stored. The relationship between the TS ID and the last updated cell ID of a TS is stored in VLR-T. When an MS not riding on a TS moves between cells, the MS updates its location information whenever the number of crossed cells from the last updated cell exceeds a predefined movement threshold for an MS.

**Figure 4.**
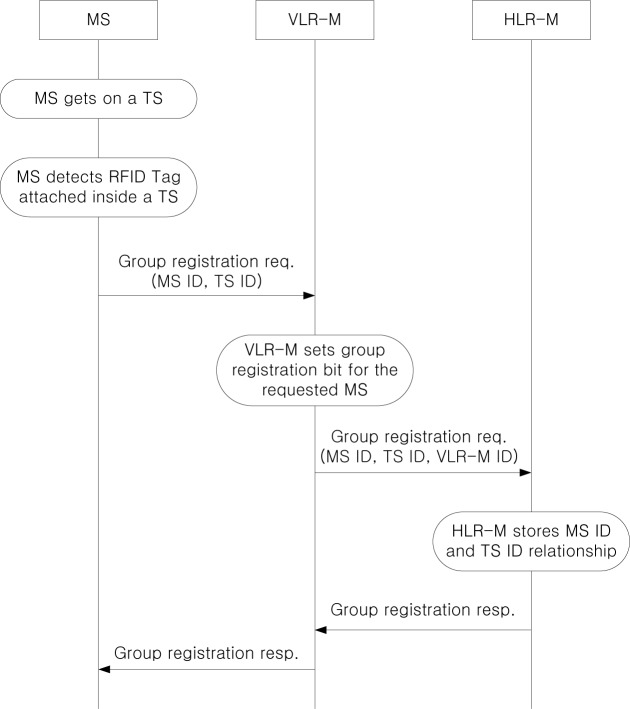
Group registration procedure.

Figure 4 shows the group registration procedure when an MS gets on a TS. If an MS gets on a TS, the RFID reader attached to the MS detects the RFID tag attached to the TS, and the MS knows that it is within a TS. Then, the MS sends a group registration request to the VLR-M, and VLR-M sets group registration bit for the requested MS. VLR-M forwards the group registration request to HLR-M, and HLR-M stores the relationship between the MS ID and the TS ID. After successful group registration, the group registration response message is delivered to the MS via VLR-M. Although base station (BS) and mobile switching center (MSC) are involved in communication between MS and VLR-M, we omit them since the main focus of this paper is to analyze the management of location information of MSC in VLR-M and HLR-M, and thus BS and MSC are not directly related.

**Figure 5.**
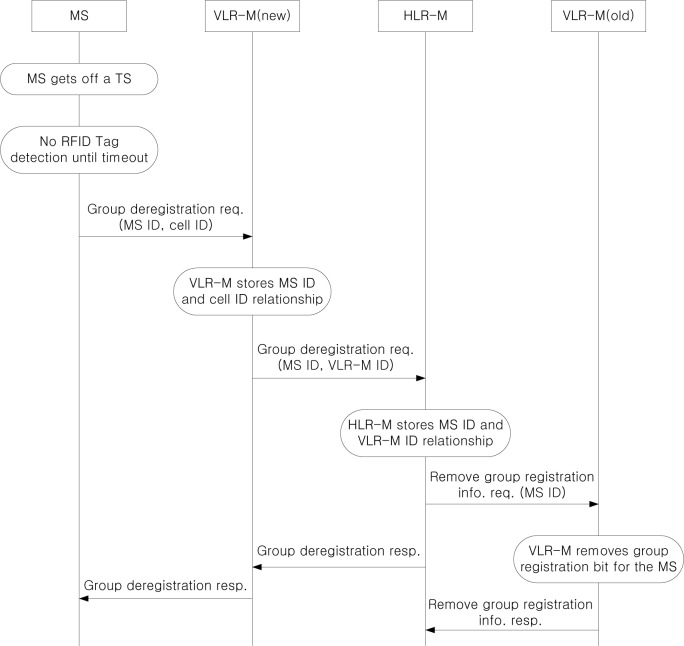
Group deregistration procedure.

Figure 5 shows group deregistration procedure when an MS gets off a TS, where an MS is managed by different VLR-M from the VLR-M where the MS has performed group registration, *i.e.*, VLR-M(new), due to the movement of an MS. If an MS gets off a TS, the RFID reader attached to the MS then no longer detects the RFID tag attached to the TS. If a predefined timer expires and no RFID tag is detected, the MS knows that it is outside the TS. Then, the MS sends a group deregistration request to VLR-M(new). Since this MS should be managed in individual location management, the VLR-M(new) stores MS ID and cell ID relationship. By doing this, an individual location update is implicitly performed during group deregistration procedure. After that, VLR-M(new) forwards the group deregistration request to HLR-M, and HLR-M stores the relationship between the MS ID and the VLR-M ID. After successful group deregistration, the group deregistration response message is delivered to the MS via VLR-M(new). In order to remove stale group registration information of the MS, the HLR-M sends the request to remove the group registration information to VLR-M(old) and the VLR-M(old) sends the response to HLR-M after the successful removal.

**Figure 6.**
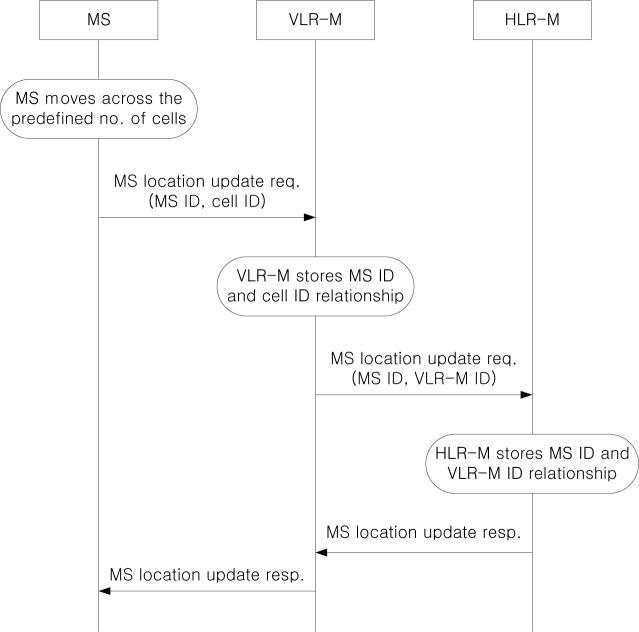
MS location update procedure.

Figure 6 shows the MS location update procedure. If an MS moves across the predefined number of cells from the last updated cell, it sends a location update request to VLR-M. Then, VLR-M stores the relationship between the MS ID and the cell ID, and forwards the location update request to HLR-M. HLR-M stores the relationship between the MS ID and the VLR-M ID, and returns the location registration response to the MS via VLR-M.

**Figure 7.**
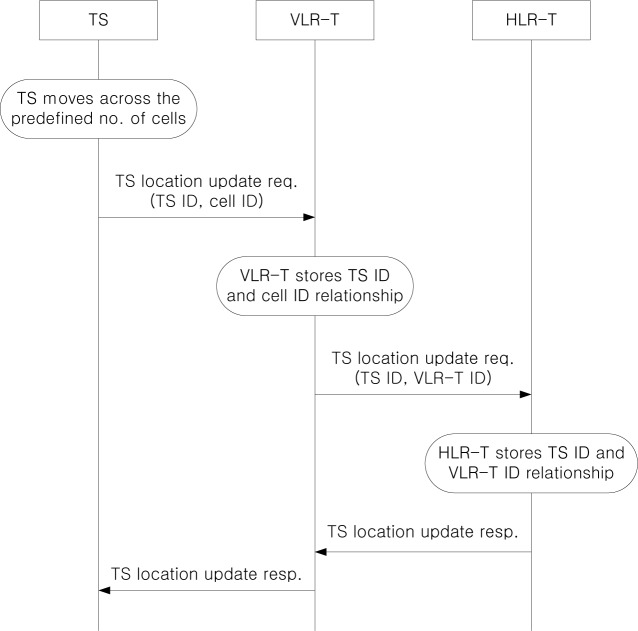
TS location update procedure.

Figure 7 shows the TS location update procedure, which is similar to that in Figure 6. If an TS moves across the predefined number of cells from the last updated cell, it sends a location update request to VLR-T. Then, VLR-T stores the relationship between the TS ID and the cell ID, and forwards the location update request to HLR-T. HLR-T stores the relationship between the TS ID and the VLR-T ID and returns the location registration response to the TS via VLR-T. We note that when an MS is riding on a TS, it does not need to update its location information, since the location information is managed by the TS.

**Figure 8.**
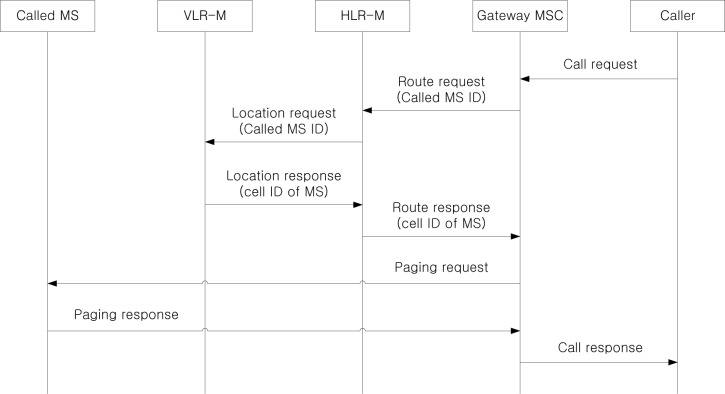
The procedure to deliver an incoming call to an MS through individual location management.

Figure 8 shows the procedure to deliver an incoming call to an MS under the individual location management, where a caller is located outside the considered network. Since the MS is not getting on a TS, the call delivery is performed based on the current VLR-M and the last updated cell information of the MS. After receiving the call request from the caller, the gateway MSC requests the route information to HLR-M based on the called MS ID. Since the MS is getting off a TS and the location information is managed by conventional individual location registration, the HLR-M knows the serving VLR-M of the MS. Therefore, it sends the location request to VLR-M, and VLR-M returns the last updated cell ID to HLR-M. Based on the cell information, the gateway MSC sends the paging request to all cells located within an area from the last updated cell of the MS to the cells of *d_MS_*-th ring, where *d_MS_* is defined as a predefined movement threshold for an MS via the serving MSC (omitted due to simplicity). If the called MS responds to the paging request, the call is established between the caller and the called MS.

**Figure 9.**
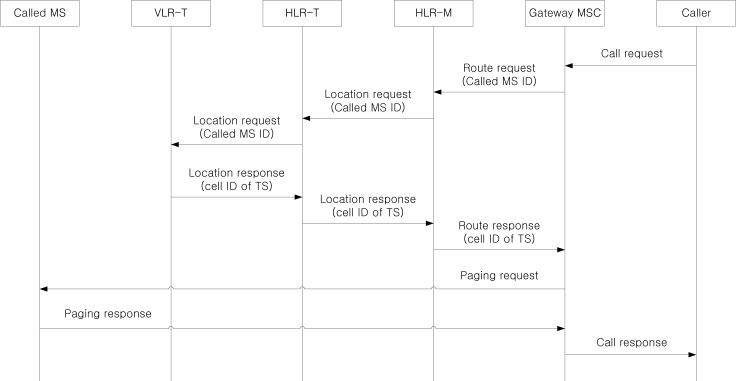
The procedure to deliver an incoming call to an MS through group location management.

Figure 9 shows the procedure to deliver an incoming call to an MS through group location management, where a caller is located outside the considered network. Since the MS is on a TS, the call delivery is performed based on the current VLR-T and the last updated cell information of the TS. After receiving the call request from the caller, the gateway MSC requests the route information to HLR-M based on the called MS ID. Since the MS is on a TS and the location information is managed by group location registration, the HLR-M does not know the serving VLR-M of the MS. Instead, it only knows the current TS ID where the MS is currently riding on. Therefore, the HLR-M sends the location request to HLR-T, and HLR-T forwards the location request to VLR-T using the stored TS ID and VLR-T ID relationship. Then, the cell ID of the TS is delivered from VLR-T to the gateway MSC via HLR-T and HLR-M. Based on the cell information, the gateway MSC sends the paging request to all cells located within an area from the last updated cell of the MS to the cells of *d_TS_*-th ring, where *d_TS_* is defined as a predefined movement threshold for a TS via serving MSC (omitted). If the called MS responds to the paging request, the call is established between the caller and the called MS.

## 3. Performance Analysis

**Figure 10.**
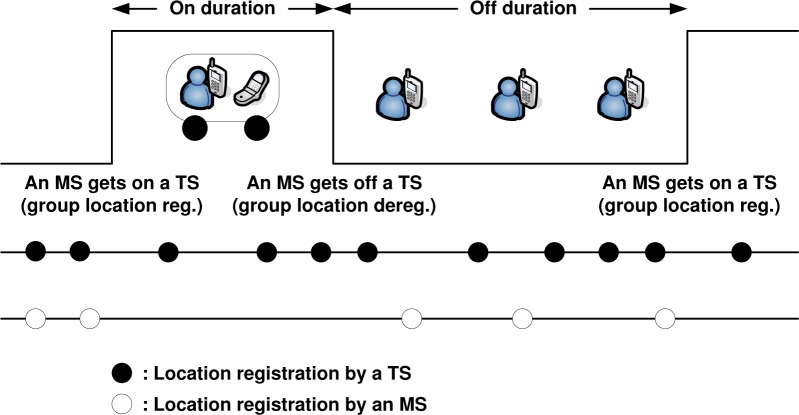
A timing diagram.

Figure 10 shows a timing diagram for the analysis of the movement-based group location management. The *on* duration denotes the time span while an MS remains on a TS, whereas the *off* duration denotes the period that an MS is not onboard a TS. The *on* and *off* durations are repeatedly interchanged, and we are interested in one consecutive pair of *on* and *off* durations. We note that under the group management, a TS always updates its location while there is any MS riding on it, but an MS updates its location only in the *off* duration and does not need to update its location in the *on* duration.

For performance analysis, we make assumptions as follows:

- The *on* duration follows an exponential distribution with parameter *λ_on_*;
- The *off* duration follows an exponential distribution with parameter *λ_off_* ;
- The cell residence time of an MS when it is riding on a TS follows a general distribution with a probability density function (pdf) $g_{\mathrm{on}}^{MS}\left(t\right)$ with mean $1/\mu_{\mathrm{on}}^{MS}$ and Laplace Transform (LT) $f_{\mathrm{on}}^{MS}\left(s\right)=\int_{0}^{\infty }e^{-st}g_{\mathrm{on}}^{MS}\left(t\right)dt$;
- The cell residence time of an MS when it is not riding on a TS follows a general distribution with a pdf $g_{\mathrm{off}}^{MS}\left(t\right)$ with mean $1/\mu_{\mathrm{off}}^{MS}$ and LT $f_{\mathrm{off}}^{MS}\left(s\right)$;
- The cell residence time of a TS during *on* duration, *i.e.*, when an observed MS is riding on the TS, follows a general distribution with a pdf $g_{\mathrm{on}}^{TS}\left(t\right)$ with mean $1/\mu_{\mathrm{on}}^{TS}$ and LT $f_{\mathrm{on}}^{TS}\left(s\right)$;
- The cell residence time of a TS during *off* duration, *i.e.*, when an observed MS is not riding on the TS, follows a general distribution with a pdf $g_{\mathrm{off}}^{TS}\left(t\right)$ with mean $1/\mu_{\mathrm{off}}^{TS}$ and LT $f_{\mathrm{off}}^{TS}\left(s\right)$;
- The call arrival rate to an MS follows a Poisson process with parameter *λ_call_*;
- The expected number of MSs riding on a TS is assumed as *N_MS_*;

We derive the number of location registration signaling messages and paging signaling messages in both individual and group location management schemes, respectively. Firstly, we consider the individual location management scheme. We note that an MS updates its location whenever the number of crossed cells from the last updated cell exceeds *d_MS_* individual location management, even when it is riding on a TS.

An MS’s cell residence time follows the distribution of $g_{\mathrm{on}}^{MS}\left(t\right)$ and $g_{\mathrm{off}}^{MS}\left(t\right)$, depending on whether the MS is riding on a TS or not, respectively. The probability that an MS moves across *K* cells during the *on* duration, $\alpha_{\mathrm{on}}^{MS}\left(K\right)$, can be derived as follows, using the results in [27,28]:

(1) $$\alpha_{\mathrm{on}}^{MS}\left(K\right)=\{\begin{array}{ll} 1-\frac{1}{\theta_{\mathrm{on}}^{MS}}\left[1-f_{\mathrm{on}}^{MS}\left(\lambda_{\mathrm{on}}\right)\right], & K=0,\\ \frac{1}{\theta_{\mathrm{on}}^{MS}}{\left[1-f_{\mathrm{on}}^{MS}\left(\lambda_{\mathrm{on}}\right)\right]}^{2}{\left[f_{\mathrm{on}}^{MS}\left(\lambda_{\mathrm{on}}\right)\right]}^{K-1}, & K>0. \end{array}$$

where $\theta_{\mathrm{on}}^{MS}$ is defined by $\lambda_{\mathrm{on}}/\mu_{\mathrm{on}}^{MS}$. Similarly, the probability that an MS moves across *K* cells during *off* duration, $\alpha_{\mathrm{off}}^{MS}\left(K\right)$, can be derived as follows:

(2) $$\alpha_{\mathrm{off}}^{MS}\left(K\right)=\{\begin{array}{ll} 1-\frac{1}{\theta_{\mathrm{off}}^{MS}}\left[1-f_{\mathrm{off}}^{MS}\left(\lambda_{\mathrm{on}}\right)\right], & K=0,\\ \frac{1}{\theta_{\mathrm{off}}^{MS}}{\left[1-f_{\mathrm{off}}^{MS}\left(\lambda_{\mathrm{on}}\right)\right]}^{2}{\left[f_{\mathrm{off}}^{MS}\left(\lambda_{\mathrm{on}}\right)\right]}^{K-1}, & K>0. \end{array}$$

where $\theta_{\mathrm{off}}^{MS}$ is defined by $\lambda_{\mathrm{on}}/\mu_{\mathrm{off}}^{MS}$.

Using the above results, the location registration signaling costs of an MS under the individual location management during *on* and *off* durations are derived as follows, respectively, using the results in [28]:

(3) $$\begin{array}{lll} {IUC}_{\mathrm{on}}^{MS} & = & U\sum_{i=1}^{\infty }i\sum_{j=id_{MS}}^{\left(i+1\right)d_{MS}-1}\alpha_{\mathrm{on}}^{MS}\left(j\right)\\ & = & U\sum_{i=1}^{\infty }i\sum_{j=id_{MS}}^{\left(i+1\right)d_{MS}-1}\frac{1}{\theta_{\mathrm{on}}^{MS}}{\left[1-f_{\mathrm{on}}^{MS}\left(\lambda_{\mathrm{on}}\right)\right]}^{2}{\left[f_{\mathrm{on}}^{MS}\left(\lambda_{\mathrm{on}}\right)\right]}^{j-1}\\ & = & U\sum_{i=1}^{\infty }i\frac{1-f_{\mathrm{on}}^{MS}\left(\lambda_{\mathrm{on}}\right)}{\theta_{\mathrm{on}}^{MS}}\left({\left[f_{\mathrm{on}}^{MS}\left(\lambda_{\mathrm{on}}\right)\right]}^{id_{MS}-1}-{\left[f_{\mathrm{on}}^{MS}\left(\lambda_{\mathrm{on}}\right)\right]}^{\left(i+1\right)d_{MS}-1}\right)\\ & = & U\frac{1-f_{\mathrm{on}}^{MS}\left(\lambda_{\mathrm{on}}\right)}{\theta_{\mathrm{on}}^{MS}}\left(\sum_{i=1}^{\infty }i\left({\left[f_{\mathrm{on}}^{MS}\left(\lambda_{\mathrm{on}}\right)\right]}^{id_{MS}-1}-{\left[f_{\mathrm{on}}^{MS}\left(\lambda_{\mathrm{on}}\right)\right]}^{\left(i+1\right)d_{MS}-1}\right)\right)\\ & = & U\frac{1-f_{\mathrm{on}}^{MS}\left(\lambda_{\mathrm{on}}\right)}{\theta_{\mathrm{on}}^{MS}}\frac{{\left[f_{\mathrm{on}}^{MS}\left(\lambda_{\mathrm{on}}\right)\right]}^{d_{MS}-1}}{1-{\left[f_{\mathrm{on}}^{MS}\left(\lambda_{\mathrm{on}}\right)\right]}^{d_{MS}}} \end{array}$$

(4) $$\begin{array}{lll} {IUC}_{\mathrm{off}}^{MS} & = & U\sum_{i=1}^{\infty }i\sum_{j=id_{MS}}^{\left(i+1\right)d_{MS}-1}\alpha_{\mathrm{off}}^{MS}\left(j\right)\\ & = & U\sum_{i=1}^{\infty }i\sum_{j=id_{MS}}^{\left(i+1\right)d_{MS}-1}\frac{1}{\theta_{\mathrm{off}}^{MS}}{\left[1-f_{\mathrm{off}}^{MS}\left(\lambda_{\mathrm{off}}\right)\right]}^{2}{\left[f_{\mathrm{off}}^{MS}\left(\lambda_{\mathrm{off}}\right)\right]}^{j-1}\\ & = & U\frac{1-f_{\mathrm{off}}^{MS}\left(\lambda_{\mathrm{off}}\right)}{\theta_{\mathrm{off}}^{MS}}\frac{{\left[f_{\mathrm{off}}^{MS}\left(\lambda_{\mathrm{off}}\right)\right]}^{d_{MS^{-1}}}}{1-{\left[f_{\mathrm{off}}^{MS}\left(\lambda_{\mathrm{off}}\right)\right]}^{d_{MS}}} \end{array}$$

where *U* is a unit cost of sending a location registration signaling message. Then, the location registration signaling cost per unit hour for an MS is obtained as:

(5) $$\begin{array}{lll} {IUC}^{MS} & = & \left({IUC}_{\mathrm{on}}^{MS}+{IUC}_{\mathrm{off}}^{MS}\right)/\left(1/\lambda_{\mathrm{on}}+1/\lambda_{\mathrm{off}}\right) \end{array}$$

(6) $$\begin{array}{lll} & = & U(\frac{1-f_{\mathrm{on}}^{MS}\left(\lambda_{\mathrm{on}}\right)}{\theta_{\mathrm{on}}^{MS}}\frac{{\left[f_{\mathrm{on}}^{MS}\left(\lambda_{\mathrm{on}}\right)\right]}^{d_{MS}-1}}{1-{\left[f_{\mathrm{on}}^{MS}\left(\lambda_{\mathrm{on}}\right)\right]}^{d_{MS}}}\\ & + & \frac{1-f_{\mathrm{off}}^{MS}\left(\lambda_{\mathrm{off}}\right)}{\theta_{\mathrm{off}}^{MS}}\frac{\left[f_{\mathrm{off}}^{MS}{\left(\lambda_{\mathrm{off}}\right)}^{d_{MS}-1}\right]}{1-{\left[f_{\mathrm{off}}^{MS}\left(\lambda_{\mathrm{off}}\right)\right]}^{d_{MS}}})/\left(1/\lambda_{\mathrm{on}}+1/\lambda_{\mathrm{off}}\right) \end{array}$$

If an incoming call arrives at an MS, paging is performed to all cells located within an area from the last updated cell of an MS to the cells of *d_MS_*-th ring. The paging signaling costs of an MS under the individual location management during *on* and *off* durations are derived as follows, respectively, using the results in [7,28]:

(7) $${IPC}_{\mathrm{on}}^{MS}=P\frac{\lambda_{\mathrm{call}}}{\lambda_{\mathrm{on}}}\left[1+3d_{MS}\left(d_{MS}-1\right)\right]$$

(8) $${IPC}_{\mathrm{off}}^{MS}=P\frac{\lambda_{\mathrm{call}}}{\lambda_{\mathrm{off}}}\left[1+3d_{MS}\left(d_{MS}-1\right)\right]$$

where *P* is a unit of cost sending a paging signaling message. Then, the paging signaling cost per unit hour for an MS under the individual location management is obtained as:

(9) $$\begin{array}{lll} {IPC}^{MS} & = & \left({IPC}_{\mathrm{on}}^{MS}+{IPC}_{\mathrm{off}}^{MS}\right)/\left(1/\lambda_{\mathrm{on}}+1/\lambda_{\mathrm{off}}\right)\\ & = & P\lambda_{\mathrm{call}}\left[1+3d_{MS}\left(d_{MS}-1\right)\right] \end{array}$$

Finally, the total signaling cost of an MS for individual location management is given by

(10) $$\begin{array}{lll} ITC & = & {IUC}^{MS}+{IPC}^{MS}\\ & = & U(\frac{1-f_{\mathrm{on}}^{MS}\left(\lambda_{\mathrm{on}}\right)}{\theta_{\mathrm{on}}^{MS}}\frac{{\left[f_{\mathrm{on}}^{MS}\left(\lambda_{\mathrm{on}}\right)\right]}^{d_{MS}-1}}{1-{\left[f_{\mathrm{on}}^{MS}\left(\lambda_{\mathrm{on}}\right)\right]}^{d_{MS}}}\\ & + & \frac{1-f_{\mathrm{off}}^{MS}\left(\lambda_{\mathrm{off}}\right)}{\theta_{\mathrm{off}}^{MS}}\frac{\left[f_{\mathrm{off}}^{MS}{\left(\lambda_{\mathrm{off}}\right)}^{d_{MS}-1}\right]}{1-{\left[f_{\mathrm{off}}^{MS}\left(\lambda_{\mathrm{off}}\right)\right]}^{d_{MS}}})/\left(1/\lambda_{\mathrm{on}}+1/\lambda_{\mathrm{off}}\right)\\ & + & P*\lambda_{\mathrm{call}}\left[1+3d_{MS}\left(d_{MS}-1\right)\right] \end{array}$$

Now, we consider the group location management scheme. The probability that a TS moves across *K* cells during the *on* duration, $\alpha_{\mathrm{on}}^{TS}\left(K\right)$, can be derived as follows:

(11) $$\alpha_{\mathrm{on}}^{TS}\left(K\right)=\{\begin{array}{ll} 1-\frac{1}{\theta_{\mathrm{on}}^{TS}}\left[1-f_{\mathrm{on}}^{TS}\left(\lambda_{\mathrm{on}}\right)\right], & K=0,\\ \frac{1}{\theta_{\mathrm{on}}^{TS}}{\left[1-f_{\mathrm{on}}^{TS}\left(\lambda_{\mathrm{on}}\right)\right]}^{2}{\left[f_{\mathrm{on}}^{TS}\left(\lambda_{\mathrm{on}}\right)\right]}^{K-1}, & K>0. \end{array}$$

where $\theta_{\mathrm{on}}^{TS}$ is defined by $\lambda_{\mathrm{on}}/\mu_{\mathrm{on}}^{TS}$. Similarly, the probability that an MS moves across *K* cells during the *off* duration, $\alpha_{\mathrm{on}}^{TS}\left(K\right)$, can be derived as follows:

(12) $$\alpha_{\mathrm{off}}^{TS}\left(K\right)=\{\begin{array}{ll} 1-\frac{1}{\theta_{\mathrm{off}}^{TS}}\left[1-f_{\mathrm{off}}^{TS}\left(\lambda_{\mathrm{on}}\right)\right], & K=0,\\ \frac{1}{\theta_{\mathrm{off}}^{TS}}{\left[1-f_{\mathrm{off}}^{TS}\left(\lambda_{\mathrm{on}}\right)\right]}^{2}{\left[f_{\mathrm{off}}^{TS}\left(\lambda_{\mathrm{on}}\right)\right]}^{K-1}, & K>0. \end{array}$$

where $\theta_{\mathrm{off}}^{TS}$ is defined by $\lambda_{\mathrm{off}}/\mu_{\mathrm{off}}^{TS}$.

The location registration signaling costs of a TS under the group location management during the *on* and *off* duration are derived as follows, respectively:

(13) $$\begin{array}{lll} {GUC}_{\mathrm{on}}^{TS} & = & U\sum_{i=1}^{\infty }i\sum_{j=id_{TS}}^{\left(i+1\right)d_{TS}-1}\alpha_{\mathrm{on}}^{TS}\left(j\right)\\ & = & U\sum_{i=1}^{\infty }i\sum_{j=id_{TS}}^{\left(i+1\right)d_{TS}-1}\frac{1}{\theta_{\mathrm{on}}^{TS}}{\left[1-f_{\mathrm{on}}^{TS}\left(\lambda_{\mathrm{on}}\right)\right]}^{2}{\left[f_{\mathrm{on}}^{TS}\left(\lambda_{\mathrm{on}}\right)\right]}^{j-1}\\ & = & U\frac{1-f_{\mathrm{on}}^{TS}\left(\lambda_{\mathrm{on}}\right)}{\theta_{\mathrm{on}}^{TS}}\frac{{\left[f_{\mathrm{on}}^{TS}\left(\lambda_{\mathrm{on}}\right)\right]}^{d_{TS}-1}}{1-{\left[f_{\mathrm{on}}^{TS}\left(\lambda_{\mathrm{on}}\right)\right]}^{d_{TS}}} \end{array}$$

(14) $$\begin{array}{lll} {GUC}_{\mathrm{off}}^{TS} & = & U\sum_{i=1}^{\infty }i\sum_{j=id_{TS}}^{\left(i+1\right)d_{TS}-1}\alpha_{\mathrm{off}}^{TS}\left(j\right)\\ & = & U\sum_{i=1}^{\infty }i\sum_{j=id_{TS}}^{\left(i+1\right)d_{TS}-1}\frac{1}{\theta_{\mathrm{off}}^{TS}}{\left[1-f_{\mathrm{off}}^{TS}\left(\lambda_{\mathrm{off}}\right)\right]}^{2}{\left[f_{\mathrm{off}}^{TS}\left(\lambda_{\mathrm{off}}\right)\right]}^{j-1}\\ & = & U\frac{1-f_{\mathrm{off}}^{TS}\left(\lambda_{\mathrm{off}}\right)}{\theta_{\mathrm{off}}^{TS}}\frac{{\left[f_{\mathrm{off}}^{TS}\left(\lambda_{\mathrm{off}}\right)\right]}^{d_{TS}-1}}{1-{\left[f_{\mathrm{off}}^{TS}\left(\lambda_{\mathrm{off}}\right)\right]}^{d_{TS}}} \end{array}$$

The location registration signaling cost of an MS under the group location management when the MS is not riding on a TS is derived as follows:

(15) $$\begin{array}{lll} {GUC}_{\mathrm{off}}^{MS} & = & U\sum_{i=1}^{\infty }i\sum_{j=id_{MS}}^{\left(i+1\right)d_{MS}-1}\alpha_{\mathrm{off}}^{MS}\left(j\right)\\ & = & U\sum_{i=1}^{\infty }i\sum_{j=id_{MS}}^{\left(i+1\right)d_{MS}-1}\frac{1}{\theta_{\mathrm{off}}^{MS}}{\left[1-f_{\mathrm{off}}^{MS}\left(\lambda_{\mathrm{off}}\right)\right]}^{2}{\left[f_{\mathrm{off}}^{MS}\left(\lambda_{\mathrm{off}}\right)\right]}^{j-1}\\ & = & U\frac{1-f_{\mathrm{off}}^{MS}\left(\lambda_{\mathrm{off}}\right)}{\theta_{\mathrm{off}}^{MS}}\frac{{\left[f_{\mathrm{off}}^{MS}\left(\lambda_{\mathrm{off}}\right)\right]}^{d_{MS}-1}}{1-{\left[f_{\mathrm{off}}^{MS}\left(\lambda_{\mathrm{off}}\right)\right]}^{d_{MS}}} \end{array}$$

We note that there is no location registration signaling cost of an MS under the group location management when the MS is riding on a TS, since the location registration is carried out by a TS, instead of the riding MSs. However, single group location registration and group location deregistration are performed when an MS gets on and gets off a TS, respectively. Therefore, the group registration signaling cost and the group deregistration signaling cost of an MS are obtained as follows:

(16) $${GUC}_{\mathrm{reg}}^{MS}=1$$

(17) $${GUC}_{\mathrm{dereg}}^{MS}=1$$

Then, the location registration signaling cost per unit hour for a TS under the group location management is obtained as:

(18) $$\begin{array}{lll} {GUC}^{TS} & = & \left({GUC}_{\mathrm{on}}^{TS}+{GUC}_{\mathrm{off}}^{TS}\right)/\left(1/\lambda_{\mathrm{on}}+1/\lambda_{\mathrm{off}}\right)\\ & = & U(\frac{1-f_{\mathrm{on}}^{TS}\left(\lambda_{\mathrm{on}}\right)}{\theta_{\mathrm{on}}^{TS}}\frac{{\left[f_{\mathrm{on}}^{TS}\left(\lambda_{\mathrm{on}}\right)\right]}^{d_{TS}-1}}{1-{\left[f_{\mathrm{on}}^{TS}\left(\lambda_{\mathrm{on}}\right)\right]}^{d_{TS}}}\\ & + & \frac{1-f_{\mathrm{off}}^{TS}\left(\lambda_{\mathrm{off}}\right)}{\theta_{\mathrm{off}}^{TS}}\frac{{\left[f_{\mathrm{off}}^{TS}\left(\lambda_{\mathrm{off}}\right)\right]}^{d_{TS}-1}}{1-{\left[f_{\mathrm{off}}^{TS}\left(\lambda_{\mathrm{off}}\right)\right]}^{d_{TS}}})/\left(1/\lambda_{\mathrm{on}}+1/\lambda_{\mathrm{off}}\right) \end{array}$$

Also, the location registration signaling cost per unit hour for an MS under the group location management is obtained as:

(19) $$\begin{array}{lll} GUC^{MS} & = & \left(GUC_{off}^{MS}+GUC_{reg}^{MS}+GUC_{dereg}^{MS}\right)/\left(1/\lambda_{on}+1/\lambda_{off}\right)\\ & = & U\left(2+\frac{1-f_{off}^{MS}\left(\lambda_{off}\right)}{\theta_{off}^{MS}}\frac{{\left[f_{off}^{MS}\left(\lambda_{off}\right)\right]}^{d_{MS}-1}}{1-{\left[f_{off}^{MS}\left(\lambda_{off}\right)\right]}^{d_{MS}}}\right)/\left(1/\lambda_{on}+1/\lambda_{off}\right) \end{array}$$

If an incoming call arrives at an MS, paging should be performed to all cells located within an appropriate area. If an MS is riding on a TS, paging is performed to all cells from the last updated cell of the TS to the cells of *d_TS_*-th ring. On the other hand, if an MS is not riding on a TS, paging is performed to all cells from the last updated cell of the MS to the cells of *d_MS_*-th ring. The paging signaling costs of an MS under the group location management during *on* and *off* duration are obtained as:

(20) $${GPC}_{\mathrm{on}}^{MS}=P\frac{\lambda_{\mathrm{call}}}{\lambda_{\mathrm{on}}}\left[1+3d_{TS}\left(d_{TS}-1\right)\right]$$

(21) $${GPC}_{\mathrm{off}}^{MS}=P\frac{\lambda_{\mathrm{call}}}{\lambda_{\mathrm{off}}}\left[1+3d_{MS}\left(d_{MS}-1\right)\right]$$

Then, the paging signaling cost per unit hour for an MS under the group location management is obtained as:

(22) $$\begin{array}{lll} {GPC}^{MS} & = & \left({GPC}_{\mathrm{on}}^{MS}+{GPC}_{\mathrm{off}}^{MS}\right)/\left(1/\lambda_{\mathrm{on}}+1/\lambda_{\mathrm{off}}\right)\\ & = & P(\frac{\lambda_{\mathrm{call}}}{\lambda_{\mathrm{on}}}\left[1+3d_{TS}\left(d_{TS}-1\right)\right]\\ & + & \frac{\lambda_{\mathrm{call}}}{\lambda_{\mathrm{off}}}\left[1+3d_{MS}\left(d_{MS}-1\right)\right])/\left(1/\lambda_{\mathrm{on}}+1/\lambda_{\mathrm{off}}\right) \end{array}$$

Finally, the total signaling cost of an MS and a TS under the group location management is given by:

(23) $$\begin{array}{lll} GTC & = & {GUC}^{MS}+{GUC}^{TS}/N_{MS}+GPC^{MS},\\ & = & (U(2+\frac{1-f_{\mathrm{off}}^{MS}\left(\lambda_{\mathrm{off}}\right)}{\theta_{\mathrm{off}}^{MS}}\frac{{\left[f_{\mathrm{off}}^{MS}\left(\lambda_{\mathrm{off}}\right)\right]}^{d_{MS}-1}}{1-{\left[f_{\mathrm{off}}^{MS}\left(\lambda_{\mathrm{off}}\right)\right]}^{d_{MS}}}\\ & + & \frac{1-f_{\mathrm{on}}^{TS}\left(\lambda_{\mathrm{on}}\right)}{\theta_{\mathrm{on}}^{TS}}\frac{{\left[f_{\mathrm{on}}^{TS}\left(\lambda_{\mathrm{on}}\right)\right]}^{d_{TS}-1}}{1-{\left[f_{\mathrm{on}}^{TS}\left(\lambda_{\mathrm{on}}\right)\right]}^{d_{TS}}}\\ & + & \frac{1-f_{\mathrm{off}}^{TS}\left(\lambda_{\mathrm{off}}\right)}{\theta_{\mathrm{off}}^{TS}}\frac{{\left[f_{\mathrm{off}}^{TS}\left(\lambda_{\mathrm{off}}\right)\right]}^{d_{TS}-1}}{1-{\left[f_{\mathrm{off}}^{TS}\left(\lambda_{\mathrm{off}}\right)\right]}^{d_{TS}}}/N_{MS})\\ & + & P(\frac{\lambda_{\mathrm{call}}}{\lambda_{\mathrm{on}}}\left[1+3d_{TS}\left(d_{TS}-1\right)\right]\\ & + & \frac{\lambda_{\mathrm{call}}}{\lambda_{\mathrm{off}}}\left[1+3d_{MS}\left(d_{MS}-1\right)\right]))/\left(1/\lambda_{\mathrm{on}}+1/\lambda_{\mathrm{off}}\right) \end{array}$$

where *GUC^TS^* is divided by the total number of riding MSs in a TS, since *GUC^TS^* is a signaling cost of a TS corresponding to all MSs riding on a TS and thus, it should be divided by *N_MS_* to normalize a cost for an MS.

## 4. Numerical Examples

In numerical examples, it is assumed that the cell residence time of an MS and a TS follows the Gamma distribution, since the Gamma distribution can represent other representative distributions such as exponential and Erlang distributions with appropriate parameters [28]. Also, it has the advantage that the measured cell residence time can be fitted with Gamma distribution appropriately if we have enough field data. The LT, $f_{\mathrm{on}}^{MS}\left(s\right)$, of the Gamma distribution with mean $1/\mu_{\mathrm{on}}^{MS}$ and variance $V_{\mathrm{on}}^{MS}$ is defined as [7,28]:

(24) $$f_{\mathrm{on}}^{MS}\left(s\right)=\left(\frac{\mu_{\mathrm{on}}^{MS}\gamma_{\mathrm{on}}^{MS}}{s+\mu_{\mathrm{on}}^{MS}\gamma_{\mathrm{on}}^{MS}}\right)\gamma_{\mathrm{on}}^{MS},\;\;\;\;\;\;\;\;\;\;\;\;\;\;\;\;\;\;\;\;\;\;\;\;\gamma_{\mathrm{on}}^{MS}=\frac{1}{V_{\mathrm{on}}^{MS}\mu_{\mathrm{on}}^{MS^{2}}}$$

**Figure 11.**
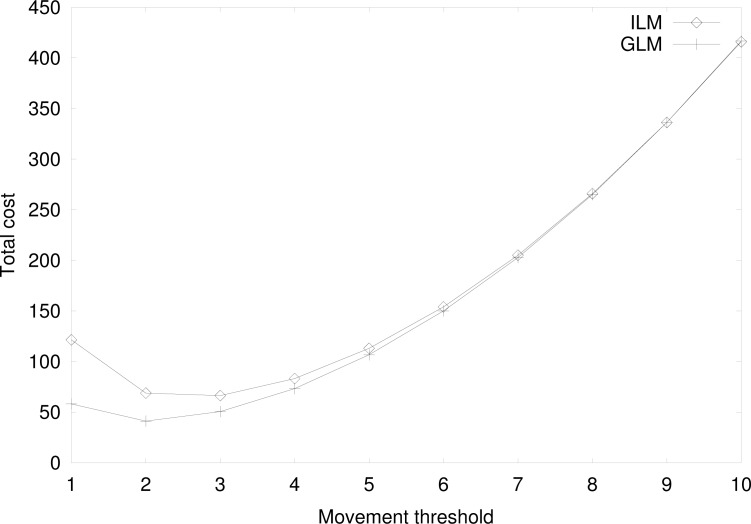
Cost comparison at varying movement thresholds when *d_MS_* = *d_TS_*.

Figure 11 shows the total cost for the individual location management (ILM) and the group location management (GLM) by varying the movement threshold with *U* = 10, *P* = 1, *λ_on_* = 1(*/hour*), *λ_off_* = 1*/*2(*/hour*), $\mu_{\mathrm{on}}^{TS}=\mu_{\mathrm{off}}^{TS}=\mu_{\mathrm{on}}^{MS}=30\left(/\mathrm{hour}\right)$, $\mu_{\mathrm{off}}^{TS}=3\left(/\mathrm{hour}\right)$, *λ_call_* = 1.5(*/hour*), *N_MS_* = 10, $\gamma_{\mathrm{on}}^{MS}=\gamma_{\mathrm{off}}^{MS}=\gamma_{\mathrm{on}}^{TS}=\gamma_{\mathrm{on}}^{TS}=1$. In Figure 11, *d_MS_* = *d_TS_* is assumed and the effect of different movement threshold values is analyzed later. For small movement threshold, the location registration signaling cost is dominant. On the other hand, the paging signaling cost is dominant for large movement threshold. Thus, as the movement threshold increases, the total cost decreases when the movement threshold is small and increases when the movement threshold is large. Since the shape of total cost is convex, there is an optimal movement threshold where the total cost is minimized. In the considered parameter set, the optimal movement threshold for the individual and the group location managements are 3 and 2, respectively. For small to medium values of movement threshold, the group location management outperforms the individual location management. At high values of movement threshold, the costs of the two location management schemes are nearly the same, since paging is a dominant factor out of total cost and both ILM and GLM have the same paging cost when *d_MS_* = *d_TS_*.

**Figure 12.**
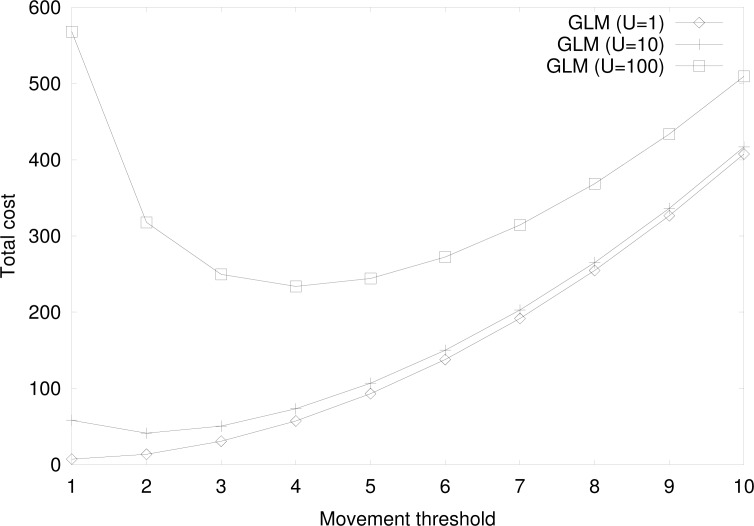
Cost comparison at varying movement thresholds when *d_MS_* = *d_TS_* for *U* = 1, 10, 100.

Figure 12 shows the total cost for the group location management by at varying movement thresholds for *U* = 1*,* 10*,* 100, with the same parameter values in Figure 11. For *U* = 1, the total cost increases as the movement threshold increases, since the paging cost is more dominant. In this case, the optimal movement threshold is 1. Otherwise, the total cost is a convex function, with the same rationale as in Figure 11, and there exists an optimal movement threshold. The optimal movement threshold value is higher for higher value of *U*.

**Figure 13.**
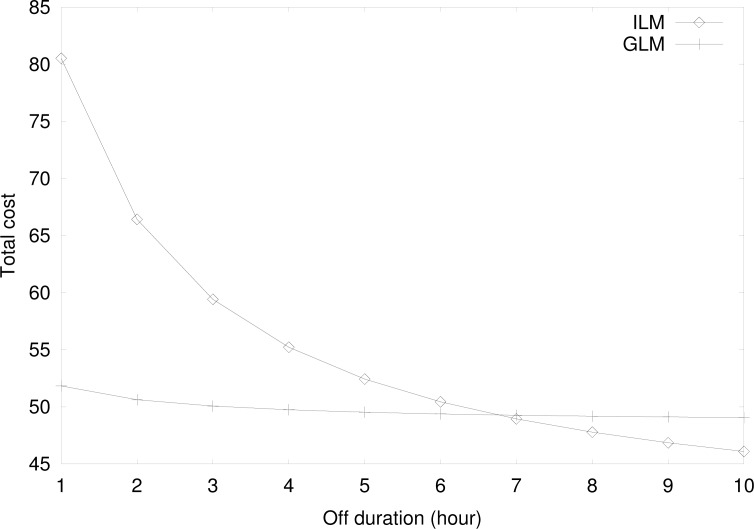
The cost for the group location management at varying *off* duration.

Figure 13 shows the total cost for group location management by varying *off* duration with the same parameter values in Figure 11, except for *d_MS_* = *d_TS_* = 3. Although the total cost for the group location management decreases as the *off* duration increases, the rate of decrease is very small because the location registration by a TS is a dominant factor in the group location management and the mobility of a TS is the same in both *on* and *off* durations. The small decrease is because the effect of the group registration and the group deregistration on the total cost decreases as the *off* duration increases. On the contrary, the total cost for the individual location management decreases significantly as the *off* duration increases. This is because the effect of high location registration of an MS riding on a TS decreases sharply as the *off* duration increases.

**Figure 14.**
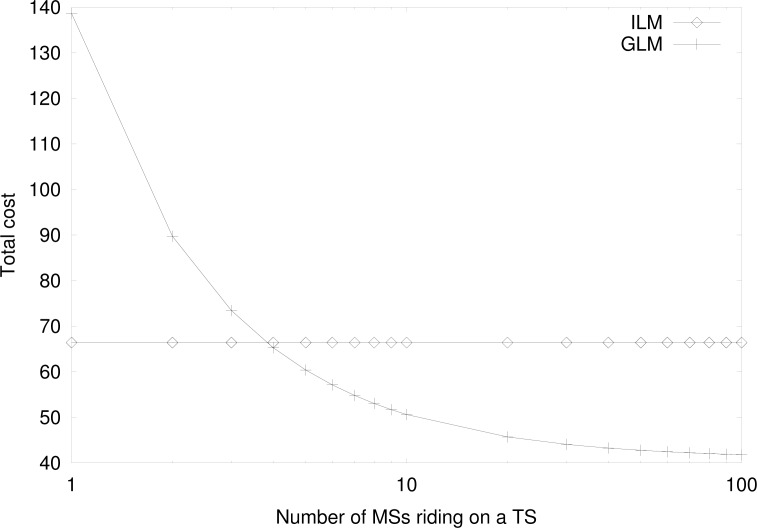
The cost for the group location management at varying number of MSs riding on a TS.

Figure 14 shows the total cost for the group location management by varying the number of MSs riding on a TS with the same parameter values in Figure 11, except for *d_MS_* = *d_TS_* = 3. Since the total cost for the individual location management does not depend on the number of MSs riding on a TS, the cost is constant. However, the total cost for the group location management decreases sharply since the effect of reducing the location registration signaling load by a TS on behalf of the riding MSs is higher for larger values of the number of MSs riding on a TS.

**Figure 15.**
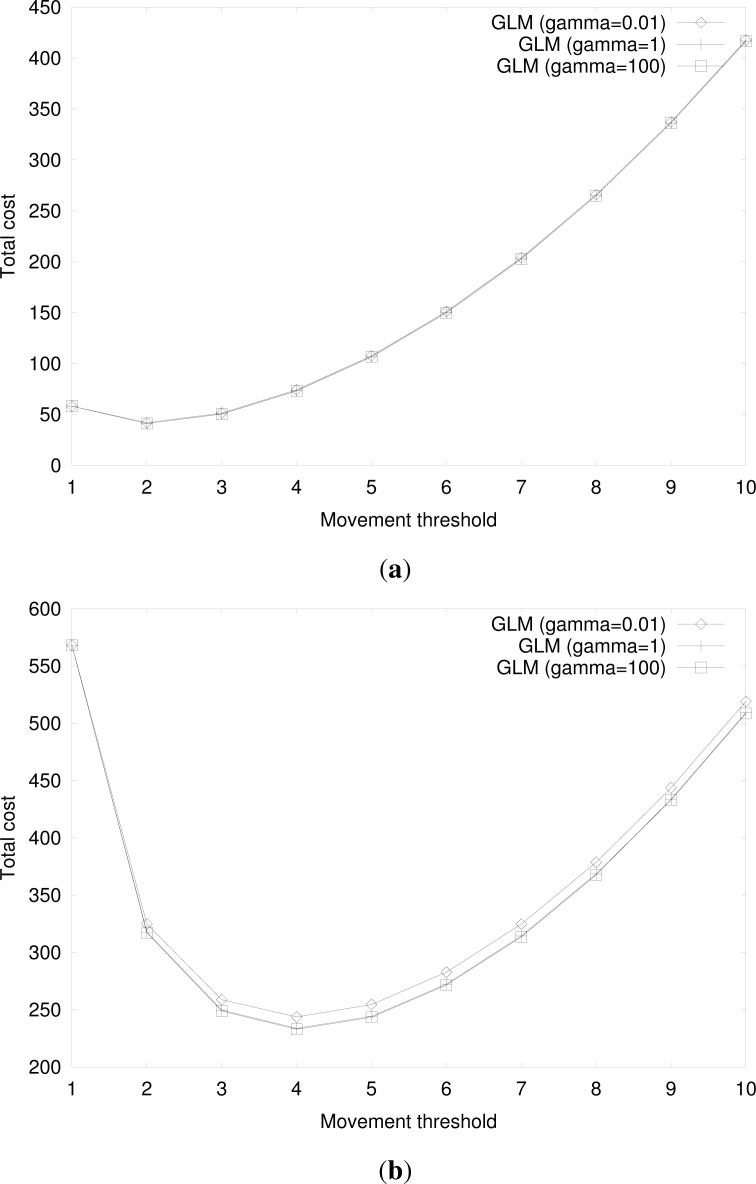
(**a**) Cost for group location management at varying movement threshold for γ = 0.01, 1, 100 and *U* = 10 when *d_MS_* = *d_TS_*. (**b**) Cost for group location management at varying movement threshold for γ = 0.01, 1, 100 and *U* = 100 when *d_MS_* = *d_TS_*.

Figure 15(a,b) shows the total cost for group location management by varying the variance of cell residence time of an MS and a TS with the same parameter values in Figure 11, for *U* = 10 and *U* = 100, respectively. In Figure 15(a), the effect of variance of cell residence time is negligible. For large values of *U*, as shown in Figure 15(b), however, the total cost with high variance of cell residence time, *i.e.*, small γ, is higher. The increase of the total cost is nevertheless slight, and thus it is shown that the variance of cell residence time does not affect the total cost of the movement-based group location management significantly. This result also verifies the validity of the mean value analysis using exponentially distributed cell residence time, as in our numerical examples.

**Figure 16.**
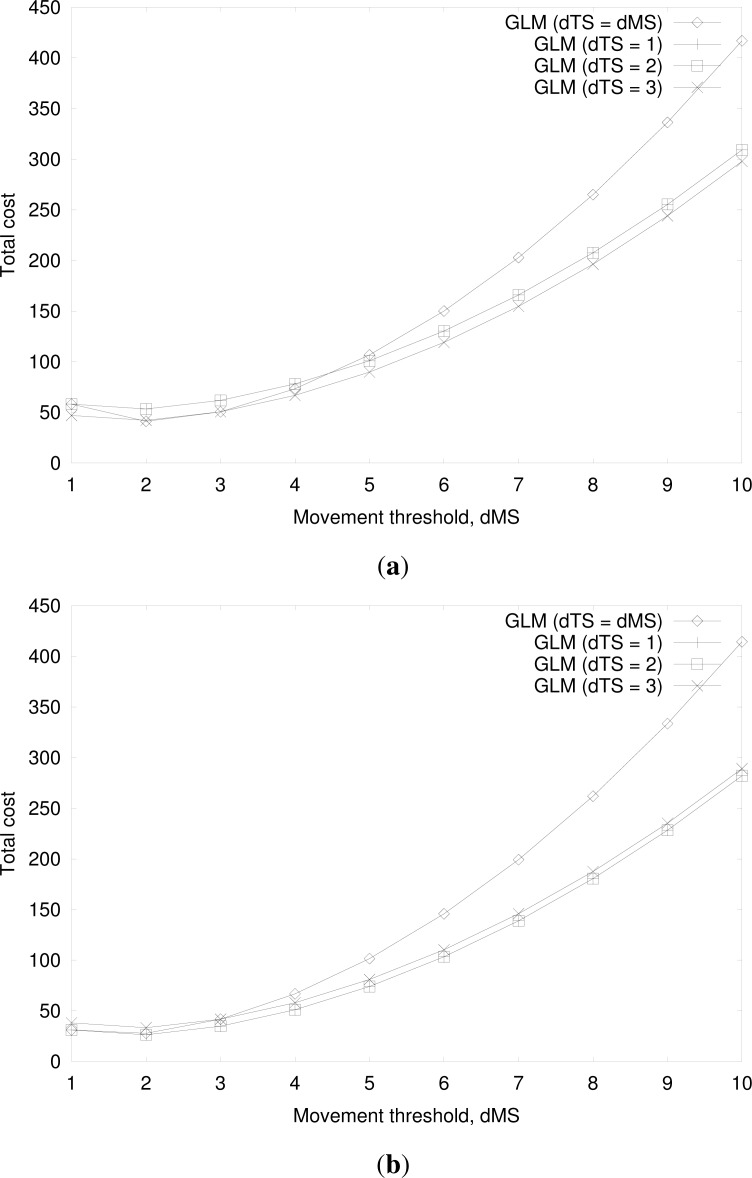
(**a**) Cost for group location management at varying movement threshold of an MS for *d_TS_* = 1, 2, 3 and *N_MS_* = 10. (**b**) Cost for group location management at varying movement threshold of an MS for *d_TS_* = 1, 2, 3 and *N_MS_* = 100.

Figure 16(a,b) show the total cost for the group location management by varying the movement threshold of an MS with the same parameter values in Figure 11, except *d_TS_* = 1*,* 2*,* 3, for *N_MS_* = 10 and *N_MS_* = 100, respectively, and compare the cost with that of the group location management for *d_MS_* = *d_TS_*. As shown in Figure 16(a,b), the group location management with small *d_TS_* has a smaller total cost than the group location management with *d_MS_* = *d_TS_* for most values of *d_MS_*, since a TS has higher mobility than the MSs and thus smaller movement threshold is more favorable. This improvement is higher for large values of *N_MS_*, since the effect of reducing the location registration signaling load is higher when the number of riding MSs in a TS is higher. This shows the validity of using separate movement threshold values for an MS and a TS, and it is concluded that the appropriate selection of *d_MS_* and *d_TS_* achieves better performance, especially for large values of *N_MS_*.

## 5. Conclusions and Future Work

In this paper, the movement-based group location management using RFID was proposed and the performance of the movement-based group location management was analyzed, from the aspect of total signaling cost. Then, the performance of the movement-based group location management was compared with that of the movement-based individual location management for varying various parameters. From the numerical examples, it can be concluded that the movement-based group location management scheme performs better than the movement-based individual location management scheme. Also, it was shown that there exists an optimal movement threshold for the movement-based group location management. Finally, an appropriate selection of separate movement threshold values for an MS and a TS achieves better performance.

In our future work, the dynamic selection of an optimal movement threshold for an MS and a TS for varying mobility and traffic characteristics will be proposed and analyzed to accommodate the varying mobility and traffic characteristics of the MSs and the TSs. Also, the validity of the proposed movement-based group location management scheme using RFID sensing will be tested.
